# Treatment outcomes and its associated factors among neonates admitted with sepsis in Hiwot Fana Comprehensive Specialized University Hospital, Harar, Ethiopia

**DOI:** 10.3389/fped.2024.1434803

**Published:** 2025-01-21

**Authors:** Betelhem Gezahegn, Ahmed Abdella, Fentahun Meseret, Ahmed Mohammed, Mulualem Keneni, Tesfaye Asfaw, Diribsa Tizazu, Assefa Desalew

**Affiliations:** ^1^ Department of Pediatrics and Child Health, Sabian General Hospital, Dire Dawa, Ethiopia; ^2^ Department of Pediatrics and Child Health, School of Medicine, College of Health and Medical Sciences, Haramaya University, Harar, Ethiopia; ^3^ Department of Pediatrics and Child Health Nursing, School of Nursing, College of Health and Medical Sciences, Haramaya University, Harar, Ethiopia

**Keywords:** neonatal sepsis, treatment outcomes, Hiwot Fana Comprehensive Specialized University Hospital, Harar, Ethiopia

## Abstract

**Background:**

Sepsis in the neonatal period is a major health challenge in neonatal medicine because of its potential for rapid progression to multi-organ dysfunction, leading to higher morbidity and mortality. Although efforts have been made to advance the outcomes of neonates admitted to hospitals, there is a paucity of data regarding neonatal sepsis treatment outcomes in the study setting. Hence, the study aimed to assess outcomes and prognostic factors of sepsis among neonatal patients admitted to the neonatal intensive care unit in Hiwot Fana Comprehensive Specialized University Hospital in Ethiopia.

**Methods:**

A facility-based cross-sectional study was conducted among 311 neonates with sepsis admitted from 1 January 2021 to 30 December 2023. Neonates were selected using systematic random sampling. Relevant data were extracted from medical records using a checklist. The data were entered into EpiData version 4.6 and analyzed using STATA version 17. Bivariable and multivariable logistic regression analyses were performed to identify factors associated with the outcome variable.

**Results:**

Eighty-four of 311 patients (27.8%) (95% CI: 22.7%–32.9%) died, while 218 (72.2%) were discharged after improvement. In the multivariable logistic regression analysis, low white blood cell (WBC) count [adjusted odds ratio (AOR) = 4.24, 95% CI: 1.5–12.5], desaturation (aOR = 3.00, 95% CI: 1.6–5.5), pre-term birth (aOR = 2.14, 95% CI: 1.1–4.0), lack of maternal antenatal care (ANC) follow-up (aOR = 2.4, 95% CI: 1.2–4.7), and chorioamnionitis (aOR = 2.8, 95% CI: 1.2–6.5) were significantly associated with neonatal sepsis mortality.

**Conclusion:**

Approximately one-quarter of patients with neonatal sepsis died. The significant prognostic factors for sepsis were found to be low WBC count, desaturation, lack of ANC visits, and chorioamnionitis. Implementing targeted therapeutic interventions and addressing these prognostic factors could improve treatment outcomes.

## Background

Neonatal sepsis is a serious infection in newborns within their first month of life, which can cause their bodies to become inflamed and weaken multiple organs (1). This can lead to septic shock, which can be life-threatening (2). Due to their immature immune systems, newborns are susceptible to infections due to exposure to bacteria during childbirth and early life (1). Neonatal sepsis is a serious condition in neonatal medicine that needs to be treated promptly (1, 2).

Neonatal sepsis is a major contributor to the global burden of neonatal mortality, with the WHO estimating that it causes about one-quarter of all neonatal deaths worldwide. Each year, it is responsible for 2.4 million newborn deaths (3, 4). The incidence of neonatal sepsis is increasing by 12.79% per year making it the third leading cause of neonatal deaths (4–6).

A systematic review conducted in 14 middle-income countries found that the incidence of neonatal sepsis was 2, 824 cases per 100,000 live births, with 17.6% of these cases resulting in death (7). Additionally, in middle- and low-income countries, neonatal mortality due to sepsis was 0.83 deaths per 1,000 live births, with the highest rates observed in South Asia and sub-Saharan African countries (8, 9). In SSA, severe forms of bacterial infections remain a leading cause of more than 1 million and 250,000 neonatal morbidity and mortality annually, respectively (10). Of these, sepsis accounts for 6.8% of neonatal deaths (11). The estimated economic burden due to these problems ranges from $10 to $469 billion (12). Neonatal mortality makes for approximately 40% of under-five deaths in Ethiopia; of these, neonatal sepsis accounts for approximately 30%–35% of neonatal deaths (13, 14).

The clinical features of neonatal sepsis can be vague and difficult to detect, making early diagnosis difficult (15). However, common signs and symptoms include drowsiness, poor feeding, temperature instability, respiratory distress, systemic inflammation, and increased heart and breathing rates (16).

The diagnosis of neonatal sepsis requires the use of comprehensive diagnostic tools, including clinical presentation, culture, complete blood cell count, and other predisposing factors (17). Other procedures such as molecular approaches, spectrometry, biomarker studies, and hematological analysis can also be used for diagnosis (16, 18). Blood cultures are considered the gold standard for diagnosis, with several adjunct tests used for clinical support (14).

The management of neonatal sepsis requires careful adherence to the latest neonatal intensive care unit (NICU) guidelines and a multidisciplinary approach (19, 20). Antimicrobial therapy targeting common pathogens associated with early- and late-onset sepsis (LOS) is typically recommended, with drug selection and duration based on culture results, clinical status, and response to treatment. For early-onset sepsis (EOS), ampicillin and gentamycin are generally administered for 7 days, while for LOSLOS, ampicillin and gentamycin are still recommended with triple antibiotics used in certain cases where the patient is critically ill (19–23).

Although the abovementioned guidelines provide a framework for management, various factors such as maternal demographics, socioeconomic issues, newborn-associated conditions, healthcare system, and institutional and professional-related factors can significantly influence the treatment outcome of neonatal sepsis (6). Antimicrobial resistance is another factor that can affect the prognosis, highlighting the importance of considering factors such as antepartum cephalosporin use and prolonged use of parenteral nutrition when selecting antibiotics (24).

Despite advancements in healthcare, neonatal sepsis remains the leading cause of morbidity and mortality among newborns worldwide, particularly in resource-limited settings. The successful management of neonatal sepsis depends on timely recognition, appropriate antimicrobial therapy, and supportive care (25). However, even with prompt intervention, the outcomes of neonatal sepsis treatment can vary widely, ranging from complete recovery to severe complications and death (26). This study focused on neonatal sepsis, as it is the most commonly identified and preventable cause of neonatal deaths in low- and middle-income countries (LMICs), contributing significantly to neonatal mortality rates (8–14). This study was intended to evaluate the progress made in accordance with the latest NICU guidelines, to support the achievement of sustainable development goals (19). So far many efforts have been made to enhance the positive outcomes of neonates admitted to hospitals in the country; however, they do not show satisfactory progress with a paucity of data specific to neonatal sepsis treatment outcomes in the study setting. Hence, this study aimed to assess the outcomes and prognostic factors of neonatal sepsis in patients admitted to the neonatal intensive care unit of Hiwot Fana Comprehensive Specialized University Hospital (HFCSUH) in Ethiopia.

## Methods and materials

### Study setting and period

The study was conducted in Harar City, located 526 km from Addis Ababa, the capital of Ethiopia. The Harari regional state has a total of seven hospitals, eight health centers, twenty-nine private clinics, twenty-six health posts, and one regional laboratory, catering to the healthcare needs of the region. This study was conducted at Hiwot Fana Comprehensive Specialized University Hospital (HFCSUH), which provides healthcare services to a population of more than six million people in the catchment area. The Pediatric and Child Health Department is one of the major units of the hospital and has four main subunits, namely, the pediatric ward, neonatal intensive care unit (NICU), outpatient units, and follow-up clinics. NICU is one of the subunits of healthcare services for newborns, with sixteen trained nurses, four working residents, and two pediatricians, and data were extracted from patient medical records between 1 and 30 January 2024.

### Study design

The study utilized an institution-based retrospective cross-sectional.

### Source and study population

The source population consisted of all neonatal patients with sepsis who were admitted to Hiwot Fana Comprehensive Specialized University Hospital, whereas the study population included all neonatal patients who were admitted with sepsis during the study period.

### Inclusion and exclusion criteria

Those neonates admitted with a confirmed diagnosis of sepsis at the NICU of HFCUSH from 1 January 2021 to 30 December 2023 were included. However, medical records missing relevant data (laboratory, clinical, and discharge outcomes) during admission were excluded from this study.

### Sample size determination and sampling procedure

A sample size of 311 neonates with sepsis was calculated using single population proportion formula [*n* = (*Z_α_*_/2_)^2^*p*(1 − *p*) / *d*^2^] where *n* is the minimum sample size required, *p* is the proportion of treatment outcome, *Z_α_*_/2_ is the value of the standard score at 95% confidence level, and *d* is the margin of error (*d* = 0.05); *p* = 24.4% (neonatal sepsis treatment outcome as a proportion) taken from a previous study conducted in Addis Ababa (27). Using the formula *n* = (1.96)^2^ × 0.244 × 0.756 (0.05)^2^ = 283 and by adding a 10% non-response rate, the final sample size was 311. The study subjects were identified using a systematic random sampling technique from the list of 1,310 neonates admitted to the NICU of the hospital from 1 January 2021 to 30 December 2023. The sampling interval (*k*) was determined by the study population (1,310 neonatal sepsis patients) divided by sample size (1,310 / 311 = 4). Data were collected for every four individuals followed by a simple random sampling technique in recruiting the first medical chart.

### Data collection method

A structured data collection tool was developed after reviewing different literature and NICU guidelines. The data abstraction format was designed to collect relevant data focusing on sociodemographic, maternal, and neonatal factors including the outcomes.

Data were collected by two trained nurses and residents after training on the objective of the study, the tool, the data collection method, and the handling of the data. Strict supervision was undertaken daily during the data collection period by one supervisor and the investigators.

Data were collected by extraction from the patients’ medical records into a structured checklist/questionnaire and checked manually for its completeness.

### Operational definition

Neonatal sepsis: diagnosed based on laboratory screening, the neonatal sepsis risk calculator, and clinical observation (28).

Early-onset sepsis: occurring within 72 h–7 days of birth, whereas LOS occurs after this time period (14, 29).

Clinical diagnosis of sepsis: based on the presence of suspected infection and clinical or laboratory evidence of infection, as well as the presence of at least two of the four systemic inflammatory response criteria (SIRC), including derangement of one of the vital signs (body temperature, heart rate, respiratory rate), desaturations, and abnormal white blood cell (WBC) counts (neutrophilia above 12,000/mm^3^ or neutropenia below 4,000/mm^3^ with 10% or more of non-segmented peripheral blood neutrophils) (30).

Low white blood cell count: defined as a white blood cell count below 1,000 cells/ml (31).

Treatment outcome: the treatment outcome of neonates admitted with sepsis was determined at the time of discharge, with two categories of outcome: either improved or not improved.

Died: a patient's medical card with death summary and other significant indicators of death on the patient database in the hospital.

Improved: a patient who has been free from signs and symptoms of neonatal sepsis and has declared as having a stable vital sign and discharge summary with a remark of improvement.

### Data quality control

Data collectors and supervisors were trained on the study objectives and data collection procedures by the principal investigator. A pretest was conducted on 5% of the sample size to ensure the validity of the data collection tool, which was then used to extract data from the patient medical charts by trained data collectors. Data quality was ensured through the use of a well-designed data abstraction tool and with continuous supervision. All collected data were checked for completeness and accuracy.

### Data processing and analysis

The collected data were coded, entered, cleaned, and stored using EpiData version 4.6 and exported to STATA 17 statistical analysis. Descriptive statistics were reported as frequency, mean, and proportion. Bivariable analysis was performed to calculate crude odds ratio and candidate variables with a *p*-value of ≤0.25 were selected for multivariable analysis to minimize the rejection of potentially relevant variables and reduce confounding issues. The goodness of fit of the model was tested using the Hosmer–Lemeshow test (>0.05). A multicollinearity test was performed to determine the correlation between the independent variables using variance inflation factors (VIF <7). The adjusted odds ratio (aOR) with 95% CI and a *p*-value of <0.05 were considered a statistically significant association.

## Results

### Admission characteristics of neonates

Among a total of 311 study participants, the analysis was performed on 302 of the 1,310 patients with a response rate of 97.1%. The mean (±SD) age of neonates was 4.77 (±5.2) days. Of the total, 245 (81.3%) were less than 1 week old at admission. Greater than half of neonates were male (58.6%) and had ≥2.5 kg (54.6%) (Table 1).

**Table 1:** Admission characteristics of neonatal sepsis patients admitted to the neonatal intensive care unit in Hiwot Fana Specialized University Hospital, Harar, Eastern Ethiopia, 2024.

Variables	Frequency	Percent (%)
Gestational week		
Pre-term	102	33.8
Term	191	63.3
Post-term	9	3.0
Age		
≤7 days	245	81.1
8–28 days	57	18.9
Sex		
Female	125	41.4
Male	177	58.6
Weight at birth		
<2.5 kg	137	45.4
≥2.5 kg	165	54.6
Resuscitated		
Yes	29	9.6
Surgery		
Yes	5	1.7
Temperature		
Normal	108	35.8
Hypo-/hyperthermic	194	64.2
RR >60 breaths/min		
Yes	187	61.9
IC or SC retraction		
Yes	98	32.5
Convulsion		
Yes	34	11.3
Fail to feed		
Yes	225	74.5

### Laboratory and clinical characteristics

**Figure 1:**
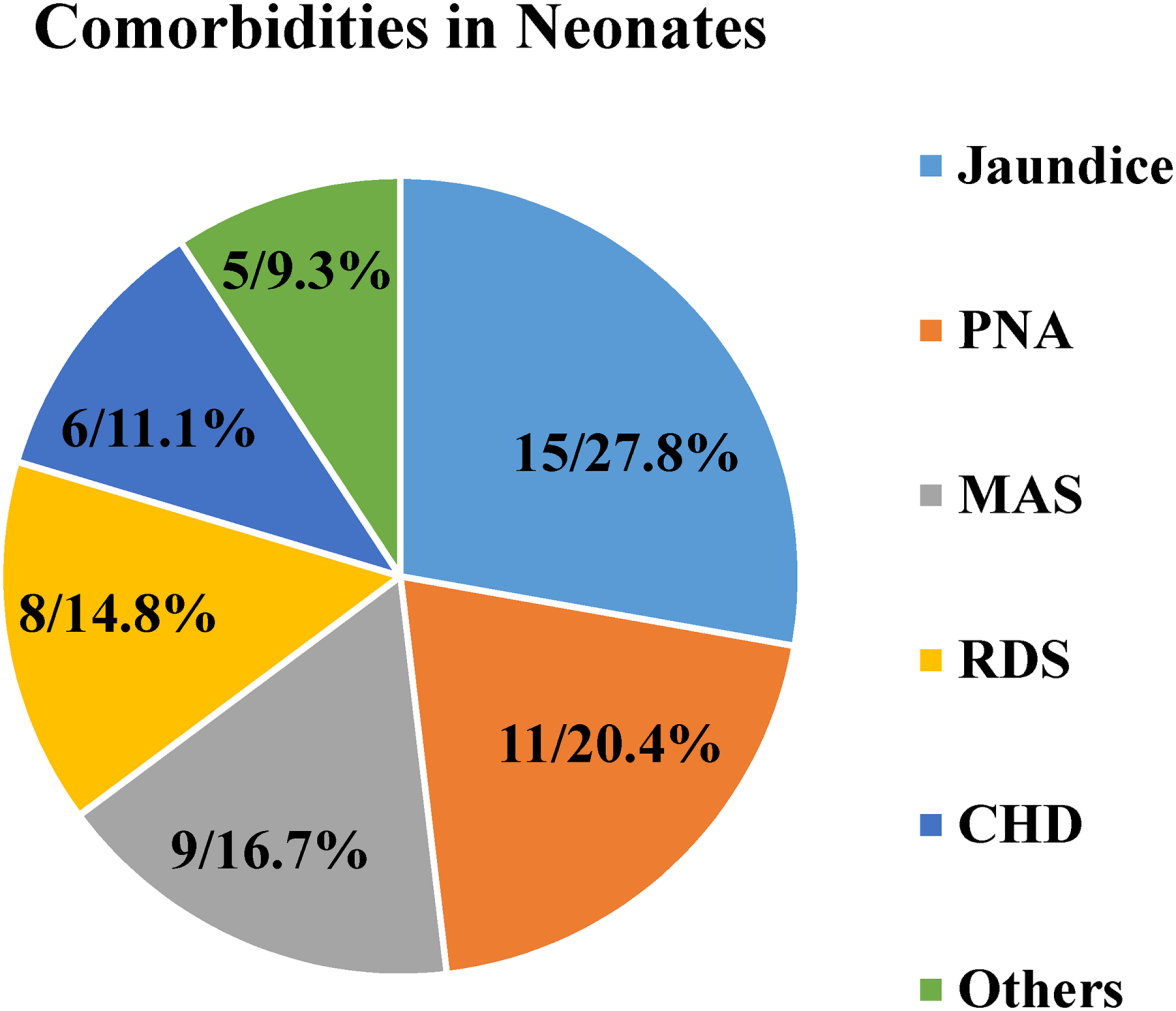
Comorbidities among neonatal sepsis patients admitted to the neonatal intensive care unit in Hiwot Fana Specialized University Hospital, Harar, Eastern Ethiopia, 2024.

Half (47.0%) of neonatal sepsis patients had abnormal WBC counts (8.0% leukopenia and 39.07% leukocytosis). The majority (62.3%) of the participants had normal platelet count, and approximately 21.9% had a platelet count of <150 × 10^3^ cells/mm^3^. Almost half (47.4%) of them had <90% SO_2_. Regarding neonatal comorbidity, 54 (17.9%) of them had identified comorbidities (Table 2). As shown in Figure 1, jaundice was the predominant (15, 27.8%) comorbidity among neonatal sepsis patients, followed by perinatal asphyxia (PNA; 11, 20.4%), meconium aspiration syndrome (MAS; 9, 16.7%), and respiratory distress syndrome (RDS; 8, 14.8%).

**Table 2:** Laboratory and clinical data of neonatal sepsis patients admitted to the neonatal intensive care unit in Hiwot Fana Specialized University Hospital, Harar, Eastern Ethiopia, 2024.

Variables	Frequency	Percent (%)
WBC count (cell/mm^3^)		
<5,000	24	8.0
5,000–21,000	160	53.0
>21,000	118	39.1
Platelet count (cell/mm^3^)		
<150 × 10^3^	66	21.9
150–450 × 10^3^	188	62.3
>450 × 10^3^	48	15.9
Hemoglobin level		
Low	96	31.8
Normal	206	68.2
Oxygen saturation		
<90%	143	47.4
≥90%	159	52.7
Neonatal comorbidity		
Yes	51	16.9
Shock		
Yes	20	6.6
MODS		
Yes	19	6.3
DIC		
Yes	15	5.0
AKI		
Yes	26	8.6
Meningitis		
Yes	49	16.2

### Maternal-related characteristics

Almost a quarter (23.5% and 25.5%) of mothers had no antenatal care (ANC) follow-up and premature rupture of membrane (PROM), respectively, in their current pregnancy. In 36 (11.9%) mothers, chorioamnionitis was diagnosed, while 41 (13.6%) had rupture of membrane (ROM) ≥18 h duration (Table 3).

**Table 3:** Maternal characteristics of neonatal sepsis patients admitted to the neonatal intensive care unit in Hiwot Fana Specialized University Hospital, Harar, Eastern Ethiopia, 2024.

Variables	Frequency	Percent (%)
ANC follow-up		
Yes	231	76.5
Chorioamnionitis		
Yes	36	11.9
Hypertensive disorder		
Yes	40	13.3
PROM		
Yes	77	25.5
ROM duration		
<18 h	261	86.4
≥18 h	41	13.6
Obstructed labor		
Yes	35	11.6
Labor duration		
< 6 h	104	34.4
6–12 h	154	51.0
13–24 h	36	11.9
>24 h	8	2.7
Multiple pregnancy		
Yes	19	6.3
Place of delivery		
Institution	293	97.0
Home	9	3.0
Mode of delivery		
CS	111	36.8
SVD	183	60.6
Instrumental	8	2.7

### Medication-related characteristics

**Figure 2:**
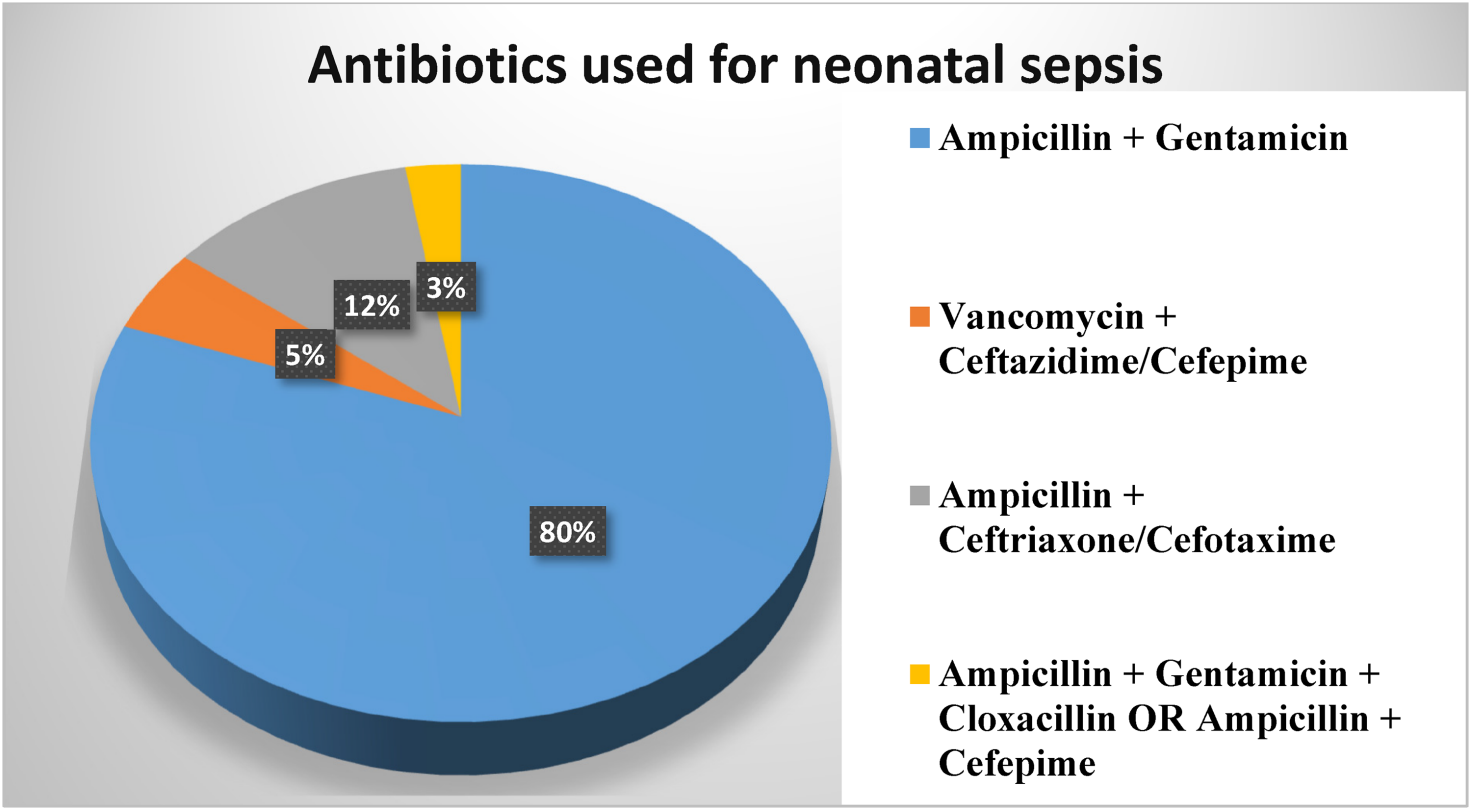
Antibiotics used in neonatal sepsis patients admitted to the neonatal intensive care unit in Hiwot Fana Specialized University Hospital, Harar, Eastern Ethiopia, 2024.

Among the study participants, 299 (99.0%) of neonatal sepsis patients were treated with dual antibiotic therapy, only 3 (1.0%) of them treated with triple antibiotics. Ampicillin and gentamicin were used to treat 243 (80.5%) patients, followed by ampicillin and third-generation cephalosporin (ceftriaxone or cefotaxime) in 36 (11.9%) patients and vancomycin with ceftazidime and cefepime in 15 (5.0%) patients (Figure 2).

### Neonatal sepsis treatment outcome

**Figure 3:**
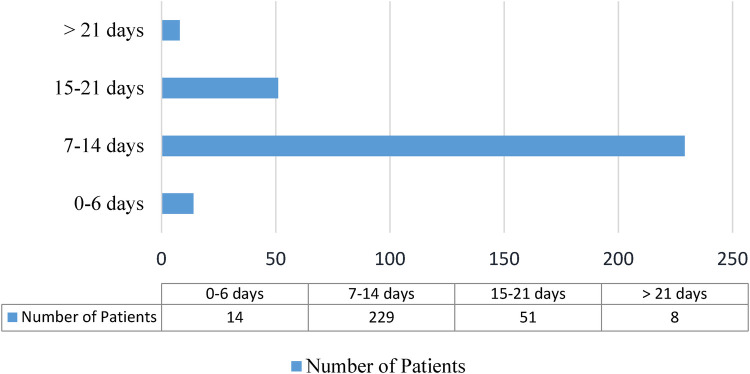
Length of hospital stay for neonatal sepsis patients admitted to the neonatal intensive care unit in Hiwot Fana Specialized University Hospital, Harar, Eastern Ethiopia, 2024.

Regarding the treatment outcome, 27.8% (95% CI: 22.7%–32.9%) of patients died, and the remaining 218 (72.2%) were discharged after improvement; three-fourths (75.8%) of neonates stayed for 7–14 days, and 59 (19.5%) of them stayed for more than fourteen days (Figure 3).

### Associated factors with neonatal sepsis treatment outcome

In the bivariable logistic regression analysis, variables such as low birth weight, gestational age, abnormal body temperature, low WBC count, abnormal platelet value, oxygen saturation, lack of ANC follow-up, prolonged ROM, meningitis, shock, MODS, and chorioamnionitis were candidates for multivariable logistic analysis. However, low WBC count, SO_2_ of <90%, no ANC visit, chorioamnionitis during pregnancy, and being pre-term neonates were statistically significant factors for neonatal sepsis mortality in multivariable analysis (Table 4).

**Table 4:** Bivariate and multivariate logistic regression analysis of factors associated with treatment outcome of neonatal sepsis patients admitted to the neonatal intensive care unit in Hiwot Fana Specialized University Hospital, Harar, Eastern Ethiopia, 2024.

Variables	Category	Treatment outcome		cOR (95% CI)	aOR (95% CI)
		Improved	Died		
Birth weight	<2.5 kg	92	45	1.6 (1.0, 2.6)	1.5 (0.8, 2.7)
	≥2.5 kg	126	39	1	1
Temperature	Normal	85	23	1	1
	Hypo-/hyperthermia	133	61	1.6 (0.9, 2.7)	1.8 (1.0, 3.5)
WBC	Low	13	11	2.9 (1.2, 7.1)	4.2 (1.5, 12.5)*
	Normal	124	36	1	1
	High	81	37	1.6 (0.9, 2.7)	1.7 (0.9, 3.3)
Platelet	Low	44	22	1.6 (0.9, 3.0)	1.5 (0.7, 3.1)
	Normal	144	44	1	1
	High	30	18	2.0 (1.0, 3.9)	1.5 (0.7, 3.4)
Oxygen saturation	<90%	85	58	3.5 (2.0, 6.0)	3.0 (1.6, 5.4)**
	≥90%	133	26	1	1
Shock	No	208	74	1	1
	Yes	10	10	2.8 (1.1, 7.0)	2.6 (0.9, 8.1)
Meningitis	No	187	66	1	1
	Yes	31	18	1.7 (0.9, 3.1)	1.3 (0.6, 2.8)
MODS	No	209	74	1	1
	Yes	9	10	3.1 (1.2, 8.0)	2.4 (0.7, 7.8)
ANC follow-up	No	40	31	2.6 (1.5, 4.6)	2.4 (1.2, 4.7)*
	Yes	178	53	1	1
Chorioamnionitis	No	203	63	1	1
	Yes	15	21	4.5 (2.2, 9.3)	2.8 (1.2, 6.5)*
ROM duration	<18 h	194	67	1	1
	≥18 h	24	17	2.1 (1.0, 4.1)	2.0 (0.9, 4.4)
Gestational week	Pre-term	64	38	2.0 (1.2, 3.4)	2.1 (1.2, 4.0)*
	Term	147	44	1	1
	Post-term	7	2	0.9 (0.2, 4.8)	0.9 (0.1,5.8)

* *P* < 0.05, whereas **P < 0.001; Hosmer–Lemeshow test = 0.62.

## Discussion

The primary aim of this study was to determine the treatment outcomes and associated factors among neonates admitted to the neonatal intensive care unit of Hiwot Fana Comprehensive Specialized University Hospital with the diagnosis of neonatal sepsis. According to this study, 27.8% (95% CI: 22.7%–32.9%) of neonatal sepsis patients died after treatment initiation. This result is analogous to studies conducted in Addis Ababa, Ethiopia, 24.4% (27); the Democratic Republic of the Congo, 21.1% (32); Nigeria, 31.8% (33); and Pakistan, 22% (34). However, the present finding accounts higher prevalence of mortality rate compared to studies conducted in the USA, 15.3% (35) and 3.3% (36); Saudi Arabia, 11.8% (37); and Switzerland, 11% (38). One potential reason for this difference could be the advanced healthcare infrastructure, availability of facilities, and high standards of care found in developed nations in comparison with the abovementioned low- and middle-income countries. Similarly in Brazil, 13% (39), lower mortality rates among neonatal sepsis patients were recorded. Another reason for the discrepancy between these findings could be associated with the fact that the study from Brazil was included only among LOSLOS neonatal patients.

On the other hand, the neonatal sepsis mortality rate in this study was lower compared to studies conducted in Tanzania, 37.1% (40); India, 44.4% (9); and Serbia, 37.5% (41). This disparity could be explained by a difference in the study population, data gathering methodology, and case classification. A study from Tanzania and India was conducted among neonates with culture-proven neonatal sepsis. In Serbia, only early-onset neonatal sepsis patients were recruited in the study, while in Tanzania, premature neonates were included. The reason for this disparity may also be the current government's devotion to diminishing neonatal mortality through sustainable development goals intended to be implemented in the country (42).

Regarding determinant factors, WBC count, level of oxygen saturation, pre-term neonates, mothers with no ANC follow-up, and chorioamnionitis were significantly associated with neonatal sepsis-related mortality.

In this study, neonates with low WBC count were approximately four times more likely to experience death than neonates having normal values of WBC count. This discovery is supported by other studies carried out in Pakistan (43) and Japan (44). This could be because low WBC neutropenia persevering along with draining neutrophils stored in the bone marrow is associated with poor prognosis (45). Although neutrophils in adults have a variety of well-established functions, such as chemotaxis, phagocytosis, and degranulation, neonatal neutrophils have been found to have less deformation and mobility of their cell membrane, which can impair their ability to effectively perform these functions, potentially leading to decreased effectiveness in fighting infections (46). Neutropenia could happen because of the imbalance between production and destruction of neutrophils (47). On the other way round, an increased absolute neutrophil count has resulted in a decrease in mortality among neonates with evidence of sepsis (46, 47). Other recent evidence also revealed that the levels of WBC on the day of sepsis onset are valuable indicators for predicting mortality in neonates with sepsis (48). However, no significant association was found between the neutrophil-to-lymphocyte ratio (NLR) and patient outcomes or length of stay (49).

Neonates who had low oxygen saturation/being desaturated had a higher chance of experiencing death than their counterparts with the odds of 3.0. The finding is analogous/aligns with the studies conducted in northwest Ethiopia (50), Tanzania (40), other low-income sub-Saharan African and South Asian countries (51), and the USA (52). The reason for this can be understood as the decrease in oxygen levels in the bloodstream impacting the oxygen supply to the body's tissues, such as organs and muscles, leading to cellular demise (53); organ dysfunction and death in neonatal sepsis are believed to be caused by bioenergetics failure due to inadequate oxygen supply to cells, indicating the importance of monitoring vital signs and clinical information to initiate timely treatment to prevent deaths and other complications in neonates (53). This highlights the need for the development of affordable and accessible tools for identifying oxygenation status in neonates, especially in limited resource settings where advanced monitoring technologies may not be readily available (40).

The study found that neonates born to mothers who did not receive antenatal care (ANC) during pregnancy were 2.4 times more likely to die from neonatal sepsis compared to neonates born to mothers who did receive ANC. This indicates that antenatal care is crucial in reducing the risk of neonatal sepsis and mortality. This is consistent with previous reports from Ethiopia (54). The failure to attend antenatal care appointments has an impact on neonatal mortality (55), possibly because it contributes to the neglect of other preventable factors during pregnancy (55). Programs that educate mothers on the importance of antenatal schedules and provide screening, monitoring, and treatment of maternal conditions during pregnancy could significantly reduce neonatal mortality rates due to neonatal sepsis. In fact, other evidence has shown that even one visit to a skilled provider during pregnancy can reduce the risk of neonatal mortality by 39% in sub-Saharan African countries (56). Hence, all pregnant women should receive ANC during pregnancy, which has important public health implications for neonatal mortality in the eastern region of Ethiopia.

Furthermore, neonates with sepsis who were delivered from mothers having a history of diagnosed chorioamnionitis had threefold odds of mortality rate as compared with those delivered from mothers with no history of chorioamnionitis. Similarly, an increased risk of neonatal sepsis mortality due to maternal chorioamnionitis has been reported in previous studies carried out in Tanzania (40) and Ethiopia (57). There is extensive evidence that histological and clinical chorioamnionitis is associated with neonatal sepsis, which can progress to adverse outcomes including short and long-term morbidity including death (58); the colonization of disease-causing agents in the birth canal and the transmission of these microorganisms to the newborn during labor and delivery can lead to neonatal sepsis, which can have adverse clinical outcomes (58). This could be further explained by the fact that chorioamnionitis or inflammation of the placenta and amniotic can commonly lead to pre-term birth, which can have a devastating effect on the development of almost every organ in the fetus (59). This highlights the importance of monitoring and managing maternal conditions during pregnancy, as it can prevent premature births and reduce the risk of neonatal sepsis and mortality (58, 59). As a clinical implication, all infants born to mothers with chorioamnionitis should be therefore directly admitted to the neonatal intensive care unit (NICU) for evaluation and treatment of presumed sepsis for a minimum of 48 h, regardless of clinical appearance with the possible implementation of a risk-stratification system for high-risk infants based on the EOS calculator (60).

Finally, pre-term neonates with sepsis were found to be 2.14 more likely to die as compared to term neonates with sepsis. The finding is consistent with previous studies conducted in Ethiopia, Nigeria, and other low- and middle-income countries (51, 61–63). Neonatal sepsis remains a serious problem among infants born pre-term, particularly they were found to be at a higher risk for life-long morbidities and death (64). This might be explained by the fact that premature infants are at an increased risk of developing septicemia as a complication because of deficiencies in humoral and cellular immunity (65). It is also known that pre-term neonates face abundant physiological challenges as they adjust to extrauterine life, which in turn results in striving to respond to treatments (59). In countries with very high neonatal mortality, additional deaths occur due to infections such as sepsis (66). This implies that timely, efficient, and effective antimicrobial therapy and supportive care are principal components of neonatal sepsis therapy, and implementing basic neonatal care to prevent sepsis through continuous professional training/education and quality improvement initiatives remains so important.

Overall, this study provides useful insights for clinical care, healthcare management, and further research in the areas of neonatal sepsis management and neonatal care specialization. Clinically, healthcare workers can identify prognostic factors associated with mortality among neonates with sepsis and implement appropriate interventions. Healthcare managers can use this evidence to assess and improve the quality of care provided by clinicians. Researchers can be encouraged to conduct further advanced research on this critical issue to develop effective strategies to reduce neonatal mortality due to sepsis.

## Limitation

Some limitations of this study include the inability to access detailed treatment protocol and laboratory results due to the reliance on medical records as well as the study being conducted in a single hospital, which may limit the generalizability of the findings to a wider population in the region or country.

Other potential limitations associated with the model we have used can also include sensitivity to outliers, linearity issues, and impact of irrelevant variables; however, to address these challenges, multicollinearity has been checked using VIF within consideration of removing highly correlated variables. We knob outliers through robust techniques with trough assumption analysis; we also conducted a thorough feature selection to include only relevant variables even from the very beginning/starting from tool preparation to have only relevant variables that can measure the intended outcome variable concisely.

## Conclusion

More than a quarter of neonates admitted with sepsis have died. Low WBC count, desaturation, lack of ANC visits, chorioamnionitis during pregnancy, and pre-term birth were statistically significant factors for mortality. Implementing effective strategies using targeted therapeutic interventions to improve treatment outcomes by enhancing intensive care services in neonates with sepsis is vital. Moreover, attention should be given to neonates with low WBC count, desaturation, and pre-term birth, and ensuring maternal ANC and timely chorioamnionitis treatment is highly recommended to improve the treatment outcomes.

## Data Availability

The original contributions presented in the study are included in the article/Supplementary Material; further inquiries can be directed to the corresponding author.
